# Biomechanical properties of osteoporotic rat femurs after different hormonal treatments: genistein, estradiol, and estradiol/progesterone

**DOI:** 10.1051/sicotj/2016016

**Published:** 2016-05-16

**Authors:** İbrahim Azboy, Mustafa Özkaya, Teyfik Demir, Abdullah Demirtaş, Arslan Kağan Arslan, Emin Özkul, Adnan Akcan, Tolga Tolunay

**Affiliations:** 1 Department of Orthopaedics and Traumatology, Dicle University 2100 Diyarbakır Turkey; 2 Department of Mechanical Engineering, TOBB University of Economics and Technology 06560 Ankara Turkey; 3 Department of Orthopaedics, Göztepe Training and Research Hospital 34888 İstanbul Turkey; 4 Department of Orthopaedics and Traumatology, Yenimahalle Training and Research Hospital 0637 Ankara Turkey

**Keywords:** Osteoporosis, Rat femur, Biomechanic, Genistein, Estradiol

## Abstract

*Introduction*: The purpose of the study is to compare the effects of genistein, estradiol, estradiol/progesterone combination on the bone mineral density and biomechanical properties of ovariectomized rats’ bone.

*Methods*: 50 female adult Sprague-Dawley rats were divided into five groups. Bilaterally ovaeriectomy were performed in all groups except the sham-operated group. Groups were a sham-operated group and a control group (water was given), estradiol treated group (17-β estradiol 0.015 mg/kg per day), genistein treated group (genistein 10 mg/kg per day), and an estradiol/progesterone combination group (17-β estradiol 0.015 mg/kg plus drosperinone 0.028 mg/kg per day). The water or hormones were implemented in relevant groups for eight weeks by orogasthric catheter. The bone mineral density and biomechanical properties of the femur were analyzed.

*Results*: Genistein, estradiol, and estradiol/progesterone groups increased bone mineral density significantly compared to the control group. In diaphysis and metaphysis bending test, all groups had higher peak load values than the control group. There were statistically significant differences between the estrogen/progesterone group and control group in diaphysis bending with regard to peak load. There were statistically significant differences between the estradiol and control groups in metaphysis bending with regard to peak load. In axial rotation test, all groups had higher peak torque values than the control groups.

*Conclusions*: Genistein, estradiol and estrogen/progesterone combination improved the biomechanical properties of the ovariectomized rat bone. Genistein which has less side effects may be considered as an alternative in the treatment of postmenopausal osteoporosis.

## Introduction

Osteoporosis is a systemic disease characterized by reduced bone mass and structural deterioration of bone. Osteoporosis is a public health issue that affects the population over 50 years of age and postmenopausal women [1]. Estrogen replacement therapy (ERT) is effective in reducing or reversing bone loss [2–4].

ERT is associated with a higher risk for breast, endometrial, and ovarian cancer [5–9]. Persson’s study [4] showed that replacement of Estrogen (EST) alone increased the risk of endometrial cancer. Replacement of EST with progesterone (PROG) reduced this higher risk [10–12]. Due to these side effects of long-term ERT use, the number of ERT users has fallen dramatically. Therefore, with the possible serious side effects of EST, there has been a growing interest in a substitute for EST with fewer side effects [13, 14]. Genistein (GEN), an isoflavone that is found abundantly in soybeans and their derivative food, could represent a natural alternative to ERT [15, 16]. Structurally, it resembles 17-β estradiol and, as a natural selective estrogen receptor modulator, it can positively regulate bone cell metabolism without the potentially dangerous estrogenic effects on other tissues [17].

The purpose of this study was to assess the EST, GEN, and EST/PROG treatment in bilateral ovariectomized female rats. Diaphysis and metaphysis bending and axial rotation tests were performed to determine biomechanical properties of the rat’s femur. To our knowledge, this is the first study which compares the effects of EST, EST/PROG, and GEN on biomechanical properties of the femur in ovariectomized rats.

## Materials and methods

The study is approved by the local Animal Ethical Committee and carried out at our University Health Sciences Practice and Research Center. All experiments were performed in accordance with the National Health and Medical Research Council (NHMRC) guidelines for the use of animals for scientific research.

### Animals

Fifty adult female Spraque-Dawley rats aged 12 weeks and weighing about 250–300 g were included in the study. The rats were kept at room temperature (22 ± 2 °C) with a photoperiod of 12 h light and 12 h dark cycle (07.00 am–07.00 pm). Throughout the experimental period standard pellet diet and water ad libitum were given to rats. Rats were anesthetized using ketamine (50 mg/kg; intraperitoneal; Ketalar^®^, Parke Davis, Eczacibasi, Istanbul, Turkey) and xylazine (5 mg/kg; intraperitoneal; Rompun^®^, Bayer AG, Leverkusen, Germany). Midline abdominal incision was made at the pelvic level and bilateral ovariectomy (OVX) was performed in all groups except the sham-operated group.

### Randomization and treatments

Twelve weeks after surgery the rats were randomly divided into five groups, each group including 10 rats. Water or hormones were injected in relevant groups for eight weeks by an orogastric catheter [18].

Group 1: (Sham group) and Group 2: (OVX group; control group); rats were given water once a day. Group 3: (OVX + EST group); rats received 17-β estradiol 0.015 mg/kg per day. Group 4: (OVX + GEN group); rats received GEN 10 mg/kg per day. Group 5: (OVX + EST/PROG group); rats received 17-β estradiol 0.015 mg/kg plus drosperinone 0.028 mg/kg per day [19].

After the end of the study, rats were sacrificed under general anesthesia. The uterus was weighed to confirm the success of ovariectomy. The femurs were freed from the skin, muscles, and tendons.

#### Densitometry

Freshly harvested bones were scanned using Dual-energy X-ray absorptiometry (DEXA). To ensure DEXA functionality, phantom calibration and quality assurance checks were also conducted prior to specimen scans. Bone mineral densities (BMD) of distal femur were measured using the accompanying small animal software.

### Biomechanical tests

The femurs were stored at a temperature of −40 °C. The fixation dies were prepared to embed the femur samples before the tests. The femur was held by means of a clamp and the fixation depth was adjusted. Polymethylmethacrylate (PMMA) was used to embed the femurs through the dies. In the diaphysis bending test, only one side of the femur was embedded in PMMA. In the metaphysis bending test, femurs were not embedded in PMMA. After the embedding process, the specimen was kept at −40 °C in a deepfreeze until the test to ensure the uniformity among the test samples’ preparation. Before starting the test, femur specimens were thawed at room temperature for 12 h, and physiologic serum was sprayed on the samples for hydration purposes.

#### Axial rotation test setup

**Figure 1.**
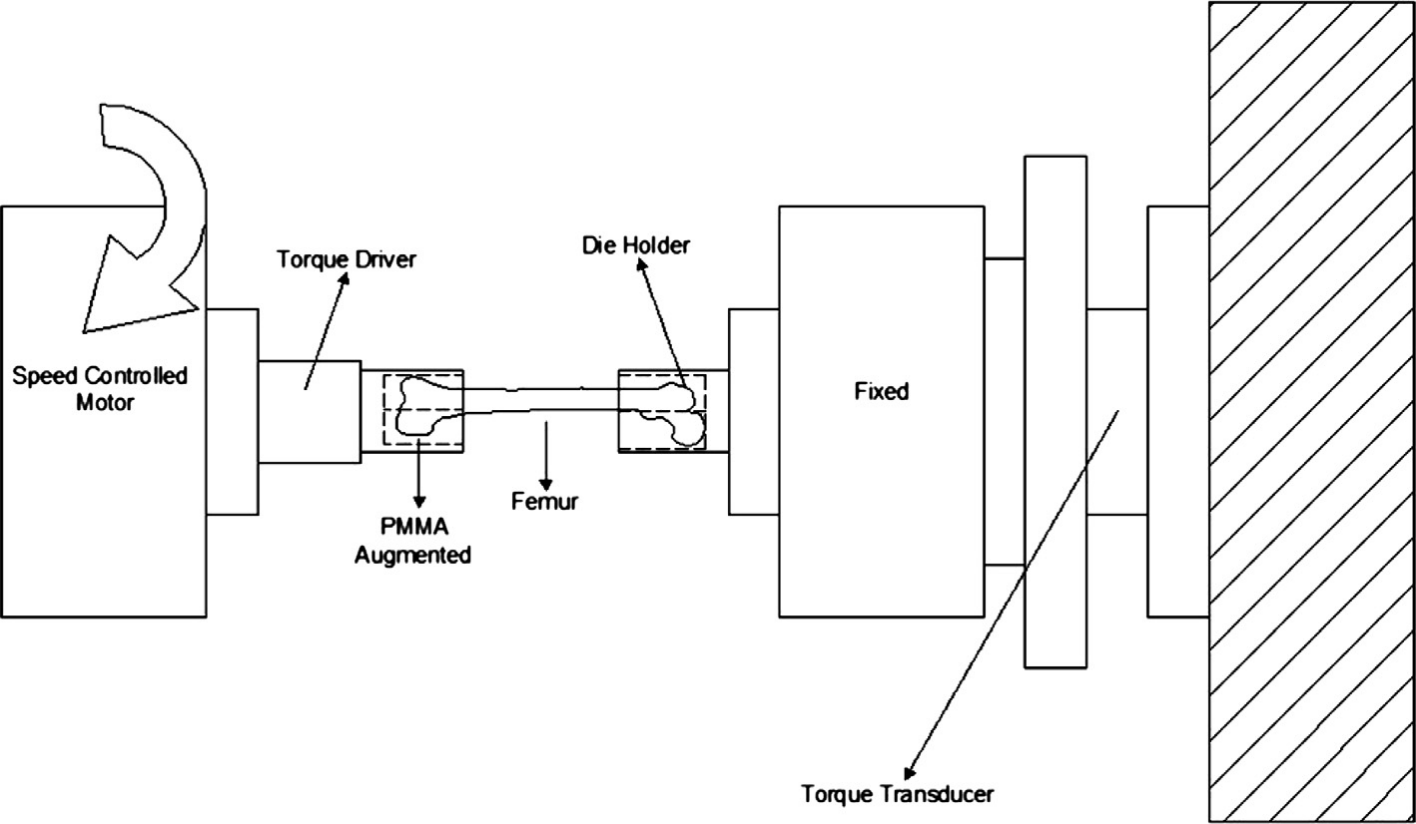
Axial rotation test setup.

To start the test, femur specimens were placed on the proper collets with the appropriate apparatus. One of the collets was fixed and contained a torque transducer. The other one was attached to the torque driver and it was allowed to rotate clockwise or counterclockwise. The Instron 55MT MicroTorsion Test machine was used for all tests. The schematic view of the test setup is shown in Figure 1. To ensure consistency, distal part of the specimens were placed in the torque driver side. After all pre-processing operations, the torque driver started to run. The torque driver twisted the femur specimens with a speed of 15 deg/min. The test maintained until the breakage of femur occurred. During the test, torque and angle of twist values were recorded with the aid of data collector.

#### Diaphysis bending test setup

**Figure 2.**
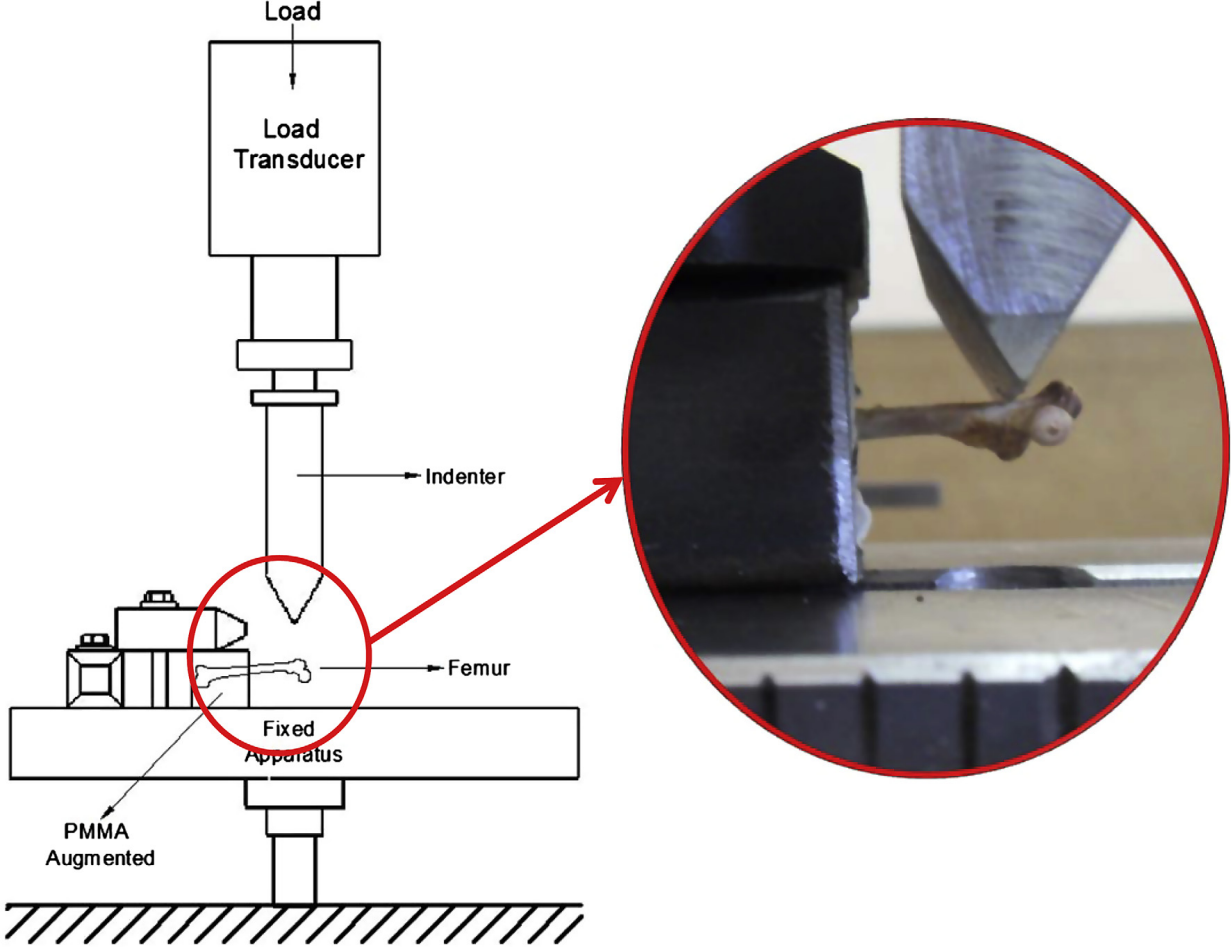
Diaphysis bending test setup.

Test specimens for the diaphysis bending test were embedded into PMMA at the distal end. Specimen was fixed to the diaphysis bending test setup as shown in Figure 2. Bending compression was applied with 2 mm/min constant crosshead speed. Failure criterion was the breakage of the rat femur. Diaphysis bending test was completed with the aid of an Instron 3300 testing machine. Yield load, yield displacement values, peak load, and peak displacement were recorded by the data collector. Stiffness values were calculated by using those values.

#### Metaphysis bending test setup

**Figure 3.**
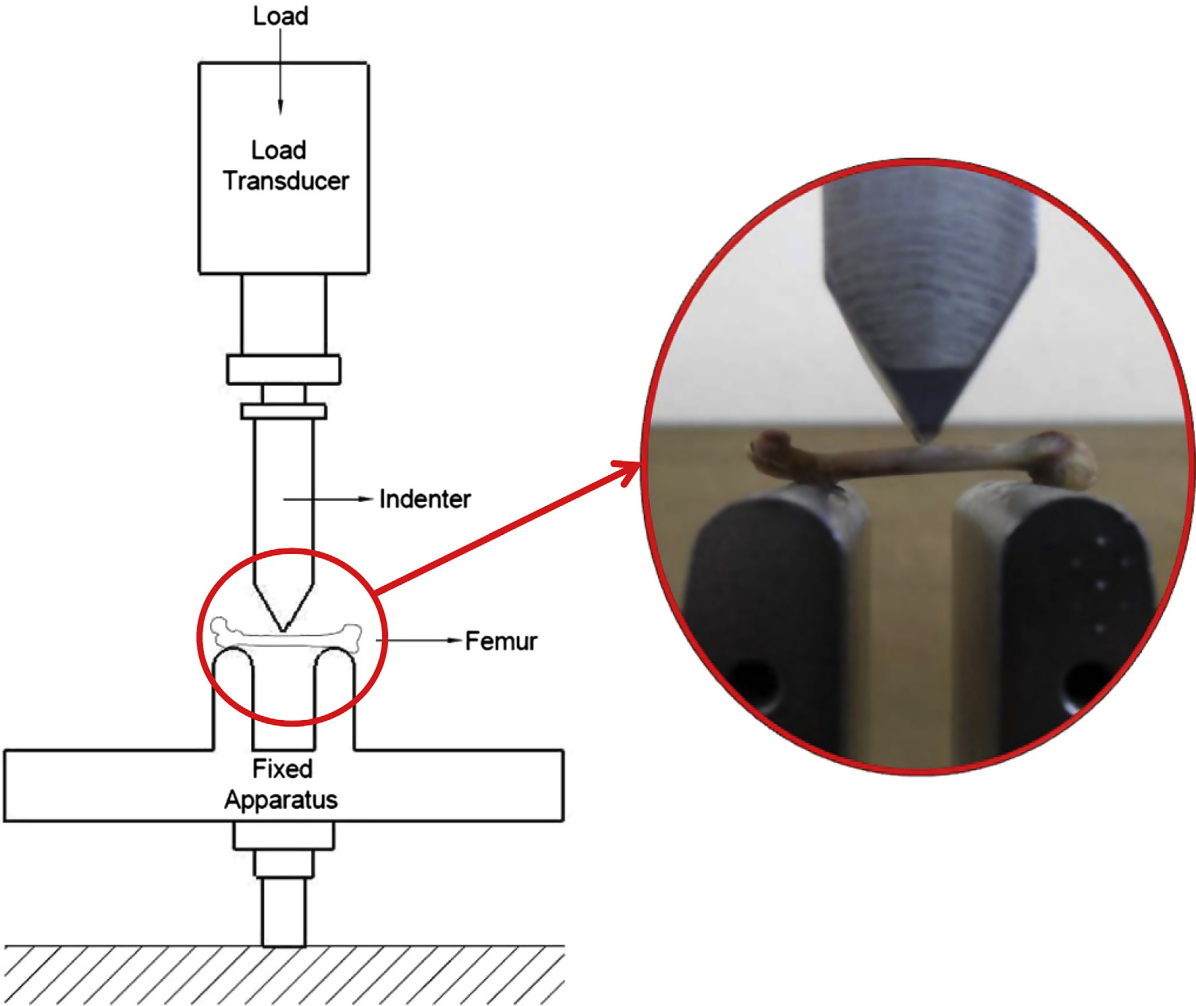
Metaphysis bending test setup.

The aim of the test was to measure the three-point bending stiffness and strength of the femur. Specimens were placed on supports at the metaphysis. The bending test setup is shown in Figure 3. The test machine, crosshead speed, and failure criterion were the same as the diaphysis bending test. Peak load and peak displacement were recorded by the data collector. Stiffness values were calculated by using those values.

All biomechanical tests were repeated five times for each test group. The values in the results tables include the mean and standard deviation of those five values.

### Statistical analyses

A one-way ANOVA was performed to ascertain the treatment effect difference between the groups at an *α* = 0.05 level. If the ANOVA test was significant, Dunnett’s test (when the variances were assumed to be equal) or Dunnett’s T3 test (when the variances were assumed to be unequal) was applied to perform post hoc pairwise comparisons at *α* = 0.05 level. For statistical analysis the Statistical Package for Social Sciences (SPSS Inc., Chicago, IL, USA) v. 18.0 for windows software was used. A *p* value < 0.05 of was considered statistically significant.

## Results

The genistein group had the highest DEXA value with 0.161 g/cm^2^, while the OVX group had the lowest value with 0.128 g/cm^2^. The bone mineral density (BMD) values for the sham, OVX + EST, and OVX + EST/PROG groups were 0.140 g/cm^2^, 0.145 g/cm^2^, and 0.143 g/cm^2^, respectively. The genistein group showed statistically significant difference compared to the other groups (Table 1).

**Table 1.** Statistical chart for bone mineral densities of groups.

Groups	*p* values
OVX vs. Sham	0.033*
OVX vs. OVX + GEN	0.001*
OVX vs. OVX + EST	0.001*
OVX vs. OVX + EST/PROG	0.007*
OVX + GEN vs. OVX + EST	0.001*
OVX + G vs. OVX + EST/PROG	0.001*
OVX + E vs. OVX + EST/PROG	0.9

* Significant difference.

OVX; Ovariectomized, GEN; Genistein, EST; Estrogen, PROG; Progesteron.

In both diaphysis and metaphysis bending tests, the sham group provided better results for the peak load and stiffness compared to the OVX groups as expected (Tables 2 and 3).

**Table 2.** Results of diaphysis bending test.

Groups	Peak load (N)		Stiffness (N/mm)	
	Mean	Sth.	Mean	Sth.
Sham	99.4	7.54	242.1	48.53
OVX	88	11.4	214.9	39.28
OVX + EST	97.4	14.28	223	66.76
OVX + EST/PROG	103	6.89	237.1	20.53
OVX + GEN	97.4	9.89	211.8	51.64

OVX; Ovariectomized, GEN; Genistein, EST; Estrogen, PROG; Progesteron.

**Table 3.** Results of metaphysis bending test.

Groups	Peak load (N)		Stiffness (N/mm)	
	Mean	Sth.	Mean	Sth.
Sham	46	10.58	68.4	17.81
OVX	34.6	4.56	30.2	7.89
OVX + EST	47.2	7.66	47.4	14.65
OVX + EST/PROG	42.4	8.53	46.6	16.58
OVX + GEN	37	5.92	50	11.5

OVX; Ovariectomized, GEN; Genistein, EST; Estrogen, PROG; Progesteron.

In diaphysis and metaphysis bending tests, the treatment groups had higher peak load values than the OVX group (Tables 2 and 3). There were statistically significant differences between the OVX + EST/PROG groups and OVX group in the diaphysis bending test regarding peak load (*p* = 0.03) but no statistically significant difference between all the treatment groups (Table 5). When comparing the stiffness values for the diaphysis bending test, the highest peak load was provided by the sham group with the value of 242.1 N/mm. There were no significant differences between the groups (Table 5).

The results for the metaphysis bending test for each group are shown in Table 4. There were statistically significant differences between the OVX + EST and OVX groups in diaphysis bending regarding peak load (*p* = 0.01) but no statistically significant difference between other groups (Table 5). Additionally, the highest stiffness value was exhibited by the sham group with 68.4 N/mm and the lowest stiffness value was 30.2 N/mm provided by the OVX group (Table 3). There was statistically significant difference between OVX + PROG and OVX groups in terms of stiffness (*p* = 0.01).

**Table 4.** Results of axial rotation test.

Groups	Peak torque (N m)		Angle at peak torque (°)	
	Mean	Sth.	Mean	Sth.
Sham	0.35	0.08	12.52	3.81
OVX	0.30	0.05	13.93	5.01
OVX + EST	0.38	0.08	13.49	3.17
OVX + EST/PROG	0.32	0.06	15.61	7.32
OVX + GEN	0.36	0.11	19.21	5.91

OVX; Ovariectomized, GEN; Genistein, EST; Estrogen, PROG; Progesteron.

**Table 5.** Statistical chart with *p* values for biomechanical tests.

Statistical chart for biomechanical tests				
	OVX vs. Sham	OVX vs. OVX + GEN	OVX vs. OVX + EST	OVX vs. OVX + EST/PROG
Diaphysis bending test				
Peak load (N)	0.099	0.201	0.283	0.036*
Stiffness (N/mm)	0.358	0.919	0.820	0.293
Metaphysis bending test				
Peak load (N)	0.058	0.493	0.013*	0.109
Stiffness (N/mm)	0.002*	0.013*	0.05	0.081
Axial rotation test				
Peak torque (N m)	0.142	0.172	0.026*	0.477
Angle at peak torque (°)	0.565	0.074	0.833	0.619

* Significant difference.

OVX; Ovariectomized, GEN; Genistein, EST; Estrogen, PROG; Progesteron.

In the axial rotation test, all groups had higher peak torque values than the OVX group (Table 4). However, significant difference was only seen between the OVX + EST and the OVX groups for peak torque values (*p* = 0.02). All treated groups exhibited the highest angles at peak torque with values of 19.21°. There were statistically significant differences between the groups (Table 5).

## Discussion

This study showed that the metaphysis and diaphysis bending and axial rotation tests in ovariectomized rat femurs suggested that the use of GEN, EST, and EST/PROG hormones improved the biomechanical properties of the bone.

Osteoporosis reduces bone mass and causes fractures that require complex surgeries [5]. Estrogen replacement therapy is effective in reducing or reversing bone loss [2, 20, 21]. However, ERT is associated with higher risk for breast, ovarian, and endometrial cancer [6–9]. GEN, an isoflavone that is found abundantly in soybeans and their derivative food, could represent a natural alternative to ERT. Epidemiologic data indicate that women ingesting high amounts of phytoESTs, particularly isoflavones in soy products, have less menopausal symptoms than those on Western diets, and consequently, bone density may be favorably influenced by phytoestrogens [6, 7]. Previous studies have shown that treatment with pure GEN aglycone increased bone mineral density (BMD) at the lumbar spine and femoral neck in postmenopausal women with no clinically significant adverse effects on the breast and uterus [13, 14]. Albertazzi has suggested that GEN aglycone would be a potentially useful, oral, bone anabolic agent [9]. In a study by Bitto et al., GEN aglycone showed a positive effect on osteoporotic bone in the ovaryectomized rat model [19]. They showed decreasing osteoclastic resorption and increasing osteoblastic formation markers. The authors also demonstrated that genistein improved the breaking strength of the femur in the three-point bending test of the femur.

In the present study, the effect of the EST, GEN, and EST/PROG on the biomechanical properties of the femur was evaluated using not only the diaphysis bending test but also metaphysis bending and axial rotation tests. In the diaphysis bending test, all treated groups had higher peak load values and stiffness than the OVX group. There were no significant differences between the OVX group and other groups for peak load. Diaphysis bending test results showed that administration of the EST, GEN, and EST/PROG increased the bone strength of the ovariectomized rats. The diaphysis bending test results also showed that there were no statistically significant differences between the OVX group and other groups for stiffness.

In the metaphysis bending test, all treated groups exhibited higher peak load and stiffness values than the OVX group. For peak load, there were no significant differences between the OVX group and other groups, except the OVX and OVX + EST treated groups (*p* = 0.01). For stiffness, the OVX + GEN treated group provided significantly higher values than the OVX group (*p* = 0.01).

In the axial rotation test, the only significant difference in the peak torque was between the OVX and OVX + EST group (*p* = 0.02). The angle at peak torque may be interpreted as the flexibility of the bone in the direction of axial rotation. There were also no significant differences in the angles at peak torque between the OVX and other groups (for all comparisons, *p* > 0.05). Although there were no significant differences for angle values, the OVX + GEN treated group may be interpreted as the most flexible one (Table 4).

In Miao’s study [22], it was noted that GEN had the restoring effect on the BMD in the bilaterally OVX rats. In the present study, the positive effect of the GEN treatment on the bone quality was also confirmed. Furthermore, GEN group had a significantly better effect on the BMD than the EST and EST/PROG groups (for both comparisons, *p* = 0.001).

This study has some limitations. Firstly, the different doses of substances were not tested; however, the food concentrations were chosen based on the results of previous studies. Secondly, the trabecular microarchitecture of the bone was not analyzed.

## Conclusions

The metaphysis and diaphysis bending and axial rotation tests suggested that the use of GEN, EST, and EST/PROG hormones improved the biomechanical properties of the ovariectomized rat bone. Although all hormone treatments improved DEXA results, GEN provided better DEXA results when compared to both EST and EST/PROG hormones. Genistein may be considered as an alternative to ERT in the treatment of postmenopausal osteoporosis.

## Conflict of interest

The authors declare no conflict of interest in relation with this paper.
